# Crystal structure of chlorido­{4,5-dimeth­oxy-2-[(2,3-η)-2-prop-2-en-1-yl]phenyl-κ*C* ^1^}(piperidine-κ*N*)platinum(II) ethanol monosolvate

**DOI:** 10.1107/S1600536814023575

**Published:** 2014-10-31

**Authors:** Peter Mangwala Kimpende, Tran Thi Da, Dinh Nguyen Huu, Luc Van Meervelt

**Affiliations:** a Chemistry Department, University of Kinshasa, Kinshasa XI BP 190, D.R. Congo; b Chemistry Department, Hanoi National University of Education, 136 – Xuan Thuy – Cau Giay, Hanoi, Vietnam; c Chemistry Department, KU Leuven, Celestijnenlaan 200F, B-3001, Leuven (Heverlee), Belgium

**Keywords:** crystal structure, platinum(II) complex, methyl­eugenol, hydrogen bonding

## Abstract

In the title compound, [Pt(C_11_H_13_O_2_)Cl(C_5_H_11_N)]·C_2_H_5_OH, the Pt^II^ cation is penta­coordinated in a distorted square-planar geometry. In the crystal, inversion dimers showing C—H⋯Cl and C—H⋯π inter­actions are further stacked in columns along the *a* axis *via* C—H⋯π inter­actions. The ethanol solvate mol­ecule inter­acts with neighbouring meth­oxy groups of methyl­eugenol through O—H⋯O hydrogen bonds.

## Abstract 2

The title platinum(II) complex shows a trigonal–bipyramidal coordination and inter­molecular C—H⋯Cl, C—H⋯π and (C/O)—H⋯O hydrogen bonds.

## Chemical context   

Methyl­eugenol or 4-allyl-1,2-di­meth­oxy­benzene (Meug, C_11_H_14_O_2_) is a natural product occurring in a number of plants such as fennel, pimento, lemongrass and nutmeg, and frequently used in perfumery and as flavouring agent (Ford *et al.*, 2000 ▶). Methyl­eugenol is used as a fruit-fly attractant in agriculture (Todd *et al.*, 2008 ▶) and in the formulation of UV absorbers, analgesics, and psychotropic drugs in medicine (Darshan & Doreswamy, 2004 ▶; Freeman & Alder, 2002 ▶). Platinum(II) complexes containing methyl­eugenol of formula [PtCl_2_(Meug)(Amine)] and deprotonated methyl­eugenol of formula [PtCl(Meug-1H)(Amine)] have been described in very few works (Da *et al.*, 2007 ▶, 2010 ▶, 2015 ▶). It is inter­esting that some of these complexes exhibit strong activities on human cancer cells KB with IC_50_ = 3.2–3.7 µg/mL (Da *et al.*, 2015 ▶). Based on these observations and prompted by the fact that one of our research areas focuses on the design and synthesis of compounds with high biological activity starting from inexpensive natural products, we have prepared the title compound [PtCl(Meug-1H)(Piperidine)] and determined its crystal structure.
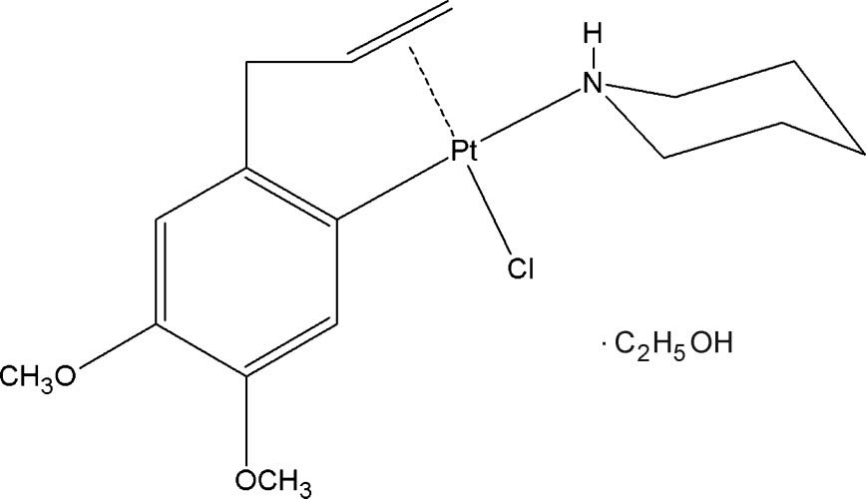


## Structural commentary   

**Figure 1:**
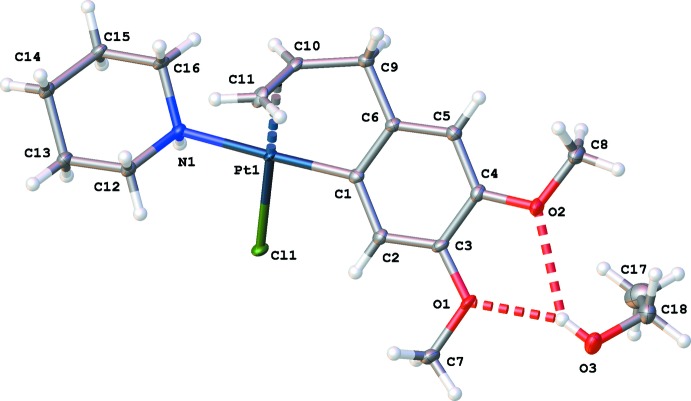
Mol­ecular structure of the title compound with displacement ellipsoids drawn at the 50% probability level and the O—H⋯O inter­actions shown as dashed lines.

In [PtCl(Meug-1H)(piperidine)], the Pt^II^ cation is penta­coordinated with Pt^II^ at the centre of a distorted square-planar geometry. The methyl­eugenol is bound with the Pt^II^ cation both at the ethyl­enic double bond and at a deprotonated benzene carbon atom (Fig. 1 ▶). The two meth­oxy groups of the methyl­eugenol part are almost in the phenyl plane, as illustrated by the torsion angles C2—C3—O1—C7 [−7.9 (6)°] and C5—C4—O2—C8 [−4.0 (6)°]. The piperidine is in the *cis* position with respect to the ethyl­enic double bond. The piperidine ring occurs in the usual chair conformation with the N1—Pt1 bond in the equatorial position. The best planes through the two six-membered rings make a dihedral angle of 24.6 (2)°. In order to avoid steric hindrance between Cl1 and the two ring systems, especially atoms C2 and C12, both rings rotate along their bond with Pt1. This is easier for the piperidine ring [resulting in a C12—N1—Pt1—Cl1 torsion angle of 70.7 (2)°] than for the phenyl ring [C2—C1—Pt1—Cl1 torsion angle of only −25.0 (4)°]. As a consequence the H12*B*⋯Cl1 distance (2.831 Å) is larger than the H2⋯Cl1 distance (2.789 Å).

## Supra­molecular features   

**Figure 2:**
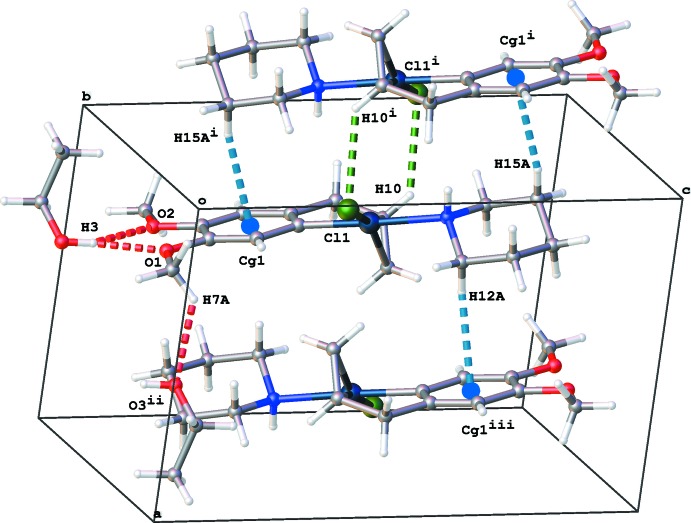
View of the crystal packing for the title compound, with O—H⋯O, C—H⋯Cl and C—H⋯π hydrogen bonds shown as red, green and blue dashed lines, respectively. *Cg*1 is the centroid of the C1–C6 ring. [Symmetry codes: (i) −*x*, −*y* + 1, −*z* + 1; (ii) −*x* + 1, −*y* + 1, −*z*; (iii) −*x* + 1, −*y* + 1, −*z* + 1.]

In the crystal packing (Fig. 2 ▶), the complex forms inversion dimers by pairs of C—H⋯Cl and C—H⋯π inter­actions (C10—H10⋯Cl1 and C15—H15*A*⋯*Cg*1 inter­actions, *Cg*1 is the centroid of the C1–C6 aromatic ring, see Table 1 ▶). These dimers are stacked in columns along [100] by C12—H12*A*⋯*Cg*1 inter­actions. The ethanol mol­ecule inter­acts by bifurcated O—H⋯O hydrogen bonds with both meth­oxy groups of methyl­eugenol and further on by C—H⋯O inter­actions to a neighboring meth­oxy group. No voids are present in the crystal packing.

## Database survey   

The Cambridge Structural Database (CSD, Version 5.35, May 2014; Groom & Allen, 2014 ▶) contains 52 1,2-di­meth­oxy­phenyl fragments in which the meth­oxy oxygen atoms inter­act simultaneously with a third oxygen atom (O⋯O distance less than the sum of the van der Waals radii). The third oxygen atom belongs in descending order to a water, alcohol, oxime or carb­oxy­lic acid, and the mean O⋯O distance is 2.916 Å. In the 690 4-substituted 1,2-di­meth­oxy­phenyl fragments present in the CSD, the majority of the C—C—O—CH_3_ torsion angles vary between −28 and +32° (only 11 torsion angles are outside this region).

## Synthesis and crystallization   

The dinuclear complex [Pt_2_Cl_2_(Meug-1H)_2_] was prepared from K[PtCl_3_(Meug)] in high yield (85%) according to Da *et al.* (2010 ▶).

The title compound was synthesized by adding a solution of 1 mmol of piperidine in 3 ml of acetone to a mixture of 408 mg (0.5 mmol) of [Pt_2_Cl_2_(Meug-1H)_2_] and 6ml of acetone. The reaction mixture was stirred at room temperature for 30 min. The obtained solution was cooled to 255 K after which the precipitate was collected and washed with Et_2_O. The yield was 320 mg (65%). The powder was dissolved in an acetone–ethanol mixture. Colourless plate-like crystals were harvested after slow evaporation of acetone at room temperature.

IR (cm^−1^): 3512 (ν_OH_ from ethanol solvate); 3247 (ν_NH_); 3060, 2946, 2838 (ν_CH_); 1581, 1557 (ν_C=C_). ^1^H NMR (δ p.p.m.; *d*_6_-acetone, Bruker Avance 500 MHz): 7.00 (1H, *s*, ^3^ *J*_PtH_ = 38 Hz, H2), 6.57 (1H, *s*, H5), 4.71 (1H, *m*, ^2^ *J*_PtH_ = 74 Hz, H10), 3.86 (1H, *d*, ^3^ *J* = 7 Hz, ^2^ *J*_PtH_ = 76 Hz, H11*A*), 3.71 (3H, *s*, methyl C7), 3.66 (3H, *s*, methyl C8), 3.61 (1H, *dd*, ^2^ *J* = 17 Hz, ^3^ *J* = 6 Hz, H9*B*), 3.57 (1H, *d*, ^3^ *J* = 13 Hz, ^2^ *J*_PtH_ = 70 Hz, H11*B*), 3.19 (1H, *t*, ^3^ *J_aa_* = 12 Hz, H1), 3.10 (1H, *d*, ^2^ *J*_ae_ = 13 Hz, H16*A*), 3.08 (1H, *d*, ^2^ *J*_ae_ = 13 Hz, H12*B*), 2.95 (1H, *qd*, ^2^ *J*_ae_ = 13 Hz, ^3^ *J*_aa_ = 12 Hz, ^3^ *J*_ae_ = 3 Hz, H12*A*), 2.93 (1H, *qd*, ^2^ *J*_ae_ = 13 Hz, ^3^ *J*_aa_ = 12 Hz, ^3^ *J*_ae_ = 3 Hz, H16*B*), 2.53 (1H, *d*, ^2^ *J* = 17 Hz, H9*A*), 1.68 (3H, *d*, ^2^ *J*_ae_ = 12 Hz, H13*A*, H14*B*, H15*B*), 1.59 (2H, *m*, H13*B*, H15*A*), 1.48 (1H, *m*, H14*A*). Calculated for [PtCl(Meug-1H)(Piperidine)]: C_16_H_24_ClNO_2_Pt, *M* = 491–495 au; found (by ESI MS, *m*/*z*): 490–494 ([*M*−H]^+^).

## Refinement   

Crystal data, data collection and structure refinement details are summarized in Table 2 ▶. All hydrogen atoms were placed in idealized positions and refined in riding mode with *U*_iso_ assigned the values to be 1.2 times those of their parent atoms (1.5 times for methyl and hydroxyl groups) with C—H distances of 0.95 (aromatic), 0.98 (meth­yl) and 0.99 Å (methyl­ene), N—H distance of 0.93 (NH) and O—H distance of 0.84 Å.

## Supplementary Material

Crystal structure: contains datablock(s) I. DOI: 10.1107/S1600536814023575/rz5134sup1.cif

Structure factors: contains datablock(s) I. DOI: 10.1107/S1600536814023575/rz5134Isup2.hkl

CCDC reference: 1031185

Additional supporting information:  crystallographic information; 3D view; checkCIF report

## Figures and Tables

**Table 1:** Hydrogen-bond geometry (, ) *Cg*1 is the centroid of the C1C6 ring.

*D*H*A*	*D*H	H*A*	*D* *A*	*D*H*A*
O3H3O1	0.84	2.10	2.869(4)	152
O3H3O2	0.84	2.47	3.158(4)	140
C10H10Cl1^i^	0.95	2.74	3.466(4)	134
C7H7*A*O3^ii^	0.98	2.59	3.276(6)	127
C15H15*A* *Cg*1^i^	0.99	2.68	3.572(5)	149
C12H12*A* *Cg*1^iii^	0.99	2.61	3.529(5)	154

Symmetry codes: (i) 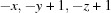; (ii) 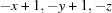; (iii) 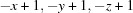.

**Table 2:** Experimental details

Crystal data	
Chemical formula	[Pt(C_11_H_13_O_2_)Cl(C_5_H_11_N)]C_2_H_6_O
*M*_r_	538.97
Crystal system, space group	Triclinic, *P* 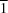
Temperature (K)	100
*a*, *b*, *c* ()	8.5280(2), 8.7520(2), 13.3309(3)
, , ()	97.905(1), 97.684(1), 99.880(1)
*V* (^3^)	958.21(4)
*Z*	2
Radiation type	Cu *K*
(mm^1^)	15.10
Crystal size (mm)	0.60 0.15 0.07

Data collection	
Diffractometer	Bruker SMART 6000
Absorption correction	Multi-scan (*SADABS*; Bruker, 2003 ▶)
*T*_min_, *T*_max_	0.107, 0.347
No. of measured, independent and observed [*I* > 2(*I*)] reflections	13448, 3533, 3410
*R*_int_	0.050
(sin /)_max_ (^1^)	0.612

Refinement	
*R*[*F* ^2^ > 2(*F* ^2^)], *wR*(*F* ^2^), *S*	0.027, 0.069, 1.08
No. of reflections	3533
No. of parameters	220
H-atom treatment	H-atom parameters constrained
_max_, _min_ (e ^3^)	1.57, 2.16

Computer programs: *SMART* and *SAINT* (Bruker, 2003 ▶), *SHELXS97* and *SHELXL97* (Sheldrick, 2008 ▶) and *OLEX2* (Dolomanov *et al.*, 2009 ▶).

## supplementary crystallographic information

### Crystal data

**Table:**

[Pt(C_11_H_13_O_2_)Cl(C_5_H_11_N)]·C_2_H_6_O	*Z* = 2
*M**_r_* = 538.97	*F*(000) = 528
Triclinic, *P*1	*D*_x_ = 1.868 Mg m^−^^3^
Hall symbol: -P 1	Cu *K*α radiation, λ = 1.54178 Å
*a* = 8.5280 (2) Å	Cell parameters from 5133 reflections
*b* = 8.7520 (2) Å	θ = 3.4–70.6°
*c* = 13.3309 (3) Å	µ = 15.10 mm^−^^1^
α = 97.905 (1)°	*T* = 100 K
β = 97.684 (1)°	Plate, colourless
γ = 99.880 (1)°	0.6 × 0.15 × 0.07 mm
*V* = 958.21 (4) Å^3^	

### Data collection

**Table:**

Bruker SMART 6000 diffractometer	3533 independent reflections
Radiation source: fine-focus sealed tube	3410 reflections with *I* > 2σ(*I*)
Crossed Globel mirrors monochromator	*R*_int_ = 0.050
ω and φ scan	θ_max_ = 70.6°, θ_min_ = 3.4°
Absorption correction: multi-scan (*SADABS*; Bruker, 2003)	*h* = −10→9
*T*_min_ = 0.107, *T*_max_ = 0.347	*k* = −10→10
13448 measured reflections	*l* = −16→16

### Refinement

**Table:**

Refinement on *F*^2^	Primary atom site location: structure-invariant direct methods
Least-squares matrix: full	Secondary atom site location: difference Fourier map
*R*[*F*^2^ > 2σ(*F*^2^)] = 0.027	Hydrogen site location: inferred from neighbouring sites
*wR*(*F*^2^) = 0.069	H-atom parameters constrained
*S* = 1.08	*w* = 1/[σ^2^(*F*_o_^2^) + (0.0436*P*)^2^] where *P* = (*F*_o_^2^ + 2*F*_c_^2^)/3
3533 reflections	(Δ/σ)_max_ = 0.002
220 parameters	Δρ_max_ = 1.57 e Å^−^^3^
0 restraints	Δρ_min_ = −2.16 e Å^−^^3^

### Special details

**Table:**

Geometry. All e.s.d.'s (except the e.s.d. in the dihedral angle between two l.s. planes) are estimated using the full covariance matrix. The cell e.s.d.'s are taken into account individually in the estimation of e.s.d.'s in distances, angles and torsion angles; correlations between e.s.d.'s in cell parameters are only used when they are defined by crystal symmetry. An approximate (isotropic) treatment of cell e.s.d.'s is used for estimating e.s.d.'s involving l.s. planes.
Refinement. Refinement of *F*^2^ against ALL reflections. The weighted *R*-factor *wR* and goodness of fit *S* are based on *F*^2^, conventional *R*-factors *R* are based on *F*, with *F* set to zero for negative *F*^2^. The threshold expression of *F*^2^ > σ(*F*^2^) is used only for calculating *R*-factors(gt) *etc*. and is not relevant to the choice of reflections for refinement. *R*-factors based on *F*^2^ are statistically about twice as large as those based on *F*, and *R*- factors based on ALL data will be even larger.

### Fractional atomic coordinates and isotropic or equivalent isotropic displacement parameters (Å^2^)

**Table:**

	*x*	*y*	*z*	*U*_iso_*/*U*_eq_	
C1	0.2696 (5)	0.6176 (4)	0.3785 (3)	0.0129 (7)	
C2	0.2869 (5)	0.5527 (4)	0.2787 (3)	0.0139 (7)	
H2	0.2772	0.4422	0.2609	0.017*	
C3	0.3180 (5)	0.6489 (5)	0.2064 (3)	0.0143 (8)	
C4	0.3316 (5)	0.8132 (4)	0.2317 (3)	0.0148 (8)	
C5	0.3171 (5)	0.8779 (4)	0.3304 (3)	0.0158 (8)	
H5	0.3271	0.9884	0.3483	0.019*	
C6	0.2877 (5)	0.7806 (4)	0.4034 (3)	0.0140 (7)	
C7	0.3414 (6)	0.4356 (4)	0.0807 (3)	0.0204 (8)	
H7A	0.4284	0.4094	0.1271	0.031*	
H7B	0.3615	0.4150	0.0099	0.031*	
H7C	0.2383	0.3710	0.0868	0.031*	
C8	0.3631 (6)	1.0615 (4)	0.1765 (3)	0.0251 (9)	
H8A	0.2608	1.0792	0.1971	0.038*	
H8B	0.3794	1.1091	0.1154	0.038*	
H8C	0.4519	1.1094	0.2325	0.038*	
C9	0.2713 (5)	0.8472 (4)	0.5106 (3)	0.0155 (7)	
H9A	0.1642	0.8765	0.5106	0.019*	
H9B	0.3548	0.9433	0.5359	0.019*	
C10	0.2902 (5)	0.7269 (4)	0.5813 (3)	0.0149 (8)	
H10	0.2109	0.7020	0.6233	0.018*	
C11	0.4226 (5)	0.6527 (4)	0.5851 (3)	0.0168 (8)	
H11A	0.5023	0.6771	0.5433	0.020*	
H11B	0.4331	0.5777	0.6296	0.020*	
C12	0.2720 (6)	0.2472 (4)	0.6279 (3)	0.0167 (8)	
H12A	0.3856	0.3012	0.6512	0.020*	
H12B	0.2655	0.1824	0.5600	0.020*	
C13	0.2216 (6)	0.1398 (5)	0.7040 (3)	0.0198 (9)	
H13A	0.2973	0.0666	0.7116	0.024*	
H13B	0.1124	0.0766	0.6773	0.024*	
C14	0.2212 (6)	0.2365 (5)	0.8087 (3)	0.0183 (8)	
H14A	0.3325	0.2908	0.8392	0.022*	
H14B	0.1806	0.1661	0.8556	0.022*	
C15	0.1132 (5)	0.3579 (5)	0.7960 (3)	0.0175 (8)	
H15A	0.0000	0.3028	0.7727	0.021*	
H15B	0.1187	0.4247	0.8631	0.021*	
C16	0.1647 (6)	0.4609 (5)	0.7188 (3)	0.0166 (8)	
H16A	0.0888	0.5336	0.7096	0.020*	
H16B	0.2732	0.5251	0.7463	0.020*	
C17	0.0871 (8)	0.8141 (7)	−0.0783 (4)	0.0431 (13)	
H17A	0.0314	0.7151	−0.1220	0.065*	
H17B	0.0305	0.8987	−0.0937	0.065*	
H17C	0.0883	0.8032	−0.0061	0.065*	
C18	0.2570 (7)	0.8527 (5)	−0.0987 (3)	0.0289 (10)	
H18A	0.2551	0.8623	−0.1720	0.035*	
H18B	0.3109	0.9554	−0.0572	0.035*	
N1	0.1688 (4)	0.3676 (4)	0.6172 (2)	0.0129 (6)	
H1	0.0645	0.3105	0.5953	0.016*	
O1	0.3355 (4)	0.5974 (3)	0.10741 (19)	0.0179 (6)	
O2	0.3593 (4)	0.8968 (3)	0.1539 (2)	0.0199 (6)	
O3	0.3470 (4)	0.7364 (4)	−0.0749 (2)	0.0259 (6)	
H3	0.3504	0.7292	−0.0124	0.039*	
Cl1	0.10276 (11)	0.26360 (9)	0.38151 (6)	0.01464 (19)	
Pt1	0.224748 (17)	0.495406 (13)	0.492434 (9)	0.01051 (9)	

### Atomic displacement parameters (Å^2^)

**Table:**

	*U*^11^	*U*^22^	*U*^33^	*U*^12^	*U*^13^	*U*^23^
C1	0.014 (2)	0.0130 (17)	0.0109 (18)	0.0030 (13)	−0.0011 (13)	−0.0001 (13)
C2	0.016 (2)	0.0157 (17)	0.0102 (17)	0.0041 (13)	−0.0001 (14)	0.0019 (13)
C3	0.017 (2)	0.0165 (18)	0.0079 (17)	0.0031 (14)	0.0011 (14)	−0.0007 (13)
C4	0.017 (2)	0.0154 (17)	0.0109 (18)	0.0013 (14)	0.0008 (14)	0.0035 (14)
C5	0.021 (2)	0.0137 (17)	0.0130 (18)	0.0057 (14)	0.0019 (15)	0.0020 (14)
C6	0.018 (2)	0.0138 (17)	0.0101 (18)	0.0037 (14)	0.0016 (14)	0.0003 (13)
C7	0.032 (2)	0.0185 (18)	0.0117 (17)	0.0053 (15)	0.0067 (15)	0.0010 (13)
C8	0.043 (3)	0.0166 (18)	0.0144 (19)	0.0030 (17)	0.0038 (16)	0.0037 (14)
C9	0.022 (2)	0.0131 (16)	0.0122 (17)	0.0061 (14)	0.0033 (14)	0.0006 (13)
C10	0.019 (2)	0.0155 (16)	0.0079 (17)	0.0007 (14)	0.0002 (13)	−0.0004 (13)
C11	0.013 (2)	0.0238 (18)	0.0117 (17)	0.0022 (14)	−0.0050 (13)	0.0040 (14)
C12	0.026 (2)	0.0146 (17)	0.0105 (17)	0.0056 (15)	0.0028 (15)	0.0024 (13)
C13	0.033 (3)	0.0168 (18)	0.0119 (19)	0.0080 (16)	0.0059 (16)	0.0031 (14)
C14	0.027 (2)	0.0189 (18)	0.0108 (18)	0.0088 (15)	0.0041 (15)	0.0036 (14)
C15	0.023 (2)	0.0224 (19)	0.0087 (17)	0.0091 (15)	0.0037 (14)	0.0022 (14)
C16	0.027 (2)	0.0171 (18)	0.0078 (17)	0.0080 (15)	0.0052 (14)	0.0008 (14)
C17	0.043 (3)	0.053 (3)	0.033 (3)	0.019 (3)	0.001 (2)	−0.002 (2)
C18	0.044 (3)	0.025 (2)	0.020 (2)	0.0132 (19)	0.0033 (18)	0.0073 (16)
N1	0.0166 (18)	0.0134 (14)	0.0081 (15)	0.0034 (12)	0.0001 (12)	0.0007 (11)
O1	0.0315 (17)	0.0161 (12)	0.0057 (12)	0.0041 (11)	0.0029 (10)	0.0011 (9)
O2	0.0338 (18)	0.0150 (12)	0.0115 (12)	0.0043 (11)	0.0038 (11)	0.0051 (10)
O3	0.0371 (19)	0.0297 (15)	0.0174 (14)	0.0148 (13)	0.0097 (12)	0.0104 (11)
Cl1	0.0205 (5)	0.0124 (4)	0.0082 (4)	0.0003 (3)	−0.0003 (3)	−0.0018 (3)
Pt1	0.01549 (13)	0.01067 (11)	0.00481 (11)	0.00278 (6)	0.00084 (7)	−0.00016 (6)

### Geometric parameters (Å, º)

**Table:**

C1—C2	1.410 (6)	C11—Pt1	2.109 (4)
C1—C6	1.396 (5)	C12—H12A	0.9900
C1—Pt1	2.014 (4)	C12—H12B	0.9900
C2—H2	0.9500	C12—C13	1.528 (5)
C2—C3	1.387 (6)	C12—N1	1.493 (5)
C3—C4	1.412 (6)	C13—H13A	0.9900
C3—O1	1.371 (5)	C13—H13B	0.9900
C4—C5	1.388 (6)	C13—C14	1.531 (5)
C4—O2	1.372 (5)	C14—H14A	0.9900
C5—H5	0.9500	C14—H14B	0.9900
C5—C6	1.400 (5)	C14—C15	1.533 (6)
C6—C9	1.501 (5)	C15—H15A	0.9900
C7—H7A	0.9800	C15—H15B	0.9900
C7—H7B	0.9800	C15—C16	1.517 (5)
C7—H7C	0.9800	C16—H16A	0.9900
C7—O1	1.423 (5)	C16—H16B	0.9900
C8—H8A	0.9800	C16—N1	1.491 (5)
C8—H8B	0.9800	C17—H17A	0.9800
C8—H8C	0.9800	C17—H17B	0.9800
C8—O2	1.425 (5)	C17—H17C	0.9800
C9—H9A	0.9900	C17—C18	1.500 (9)
C9—H9B	0.9900	C18—H18A	0.9900
C9—C10	1.521 (5)	C18—H18B	0.9900
C10—H10	0.9500	C18—O3	1.420 (6)
C10—C11	1.395 (6)	N1—H1	0.9300
C10—Pt1	2.143 (4)	N1—Pt1	2.188 (3)
C11—H11A	0.9500	O3—H3	0.8400
C11—H11B	0.9500	Cl1—Pt1	2.3289 (8)
			
C2—C1—Pt1	125.6 (3)	C12—C13—H13B	109.5
C6—C1—C2	118.6 (3)	C12—C13—C14	110.7 (3)
C6—C1—Pt1	115.7 (3)	H13A—C13—H13B	108.1
C1—C2—H2	119.8	C14—C13—H13A	109.5
C3—C2—C1	120.5 (4)	C14—C13—H13B	109.5
C3—C2—H2	119.8	C13—C14—H14A	109.8
C2—C3—C4	120.4 (4)	C13—C14—H14B	109.8
O1—C3—C2	125.0 (3)	C13—C14—C15	109.6 (3)
O1—C3—C4	114.7 (3)	H14A—C14—H14B	108.2
C5—C4—C3	119.4 (3)	C15—C14—H14A	109.8
O2—C4—C3	115.5 (3)	C15—C14—H14B	109.8
O2—C4—C5	125.1 (3)	C14—C15—H15A	109.4
C4—C5—H5	120.0	C14—C15—H15B	109.4
C4—C5—C6	120.1 (4)	H15A—C15—H15B	108.0
C6—C5—H5	120.0	C16—C15—C14	111.3 (4)
C1—C6—C5	121.1 (4)	C16—C15—H15A	109.4
C1—C6—C9	117.7 (3)	C16—C15—H15B	109.4
C5—C6—C9	121.2 (3)	C15—C16—H16A	109.1
H7A—C7—H7B	109.5	C15—C16—H16B	109.1
H7A—C7—H7C	109.5	H16A—C16—H16B	107.8
H7B—C7—H7C	109.5	N1—C16—C15	112.5 (3)
O1—C7—H7A	109.5	N1—C16—H16A	109.1
O1—C7—H7B	109.5	N1—C16—H16B	109.1
O1—C7—H7C	109.5	H17A—C17—H17B	109.5
H8A—C8—H8B	109.5	H17A—C17—H17C	109.5
H8A—C8—H8C	109.5	H17B—C17—H17C	109.5
H8B—C8—H8C	109.5	C18—C17—H17A	109.5
O2—C8—H8A	109.5	C18—C17—H17B	109.5
O2—C8—H8B	109.5	C18—C17—H17C	109.5
O2—C8—H8C	109.5	C17—C18—H18A	109.2
C6—C9—H9A	109.6	C17—C18—H18B	109.2
C6—C9—H9B	109.6	H18A—C18—H18B	107.9
C6—C9—C10	110.2 (3)	O3—C18—C17	112.2 (4)
H9A—C9—H9B	108.1	O3—C18—H18A	109.2
C10—C9—H9A	109.6	O3—C18—H18B	109.2
C10—C9—H9B	109.6	C12—N1—H1	105.2
C9—C10—H10	119.6	C12—N1—Pt1	110.7 (2)
C9—C10—Pt1	109.1 (2)	C16—N1—C12	111.2 (3)
C11—C10—C9	120.7 (4)	C16—N1—H1	105.2
C11—C10—H10	119.6	C16—N1—Pt1	118.0 (2)
C11—C10—Pt1	69.5 (2)	Pt1—N1—H1	105.2
Pt1—C10—H10	91.3	C3—O1—C7	117.2 (3)
C10—C11—H11A	120.0	C4—O2—C8	116.0 (3)
C10—C11—H11B	120.0	C18—O3—H3	109.5
C10—C11—Pt1	72.2 (2)	C1—Pt1—C10	81.39 (15)
H11A—C11—H11B	120.0	C1—Pt1—C11	86.96 (15)
Pt1—C11—H11A	107.8	C1—Pt1—N1	177.85 (13)
Pt1—C11—H11B	90.0	C1—Pt1—Cl1	94.15 (11)
H12A—C12—H12B	107.8	C10—Pt1—N1	97.56 (13)
C13—C12—H12A	109.1	C10—Pt1—Cl1	167.26 (12)
C13—C12—H12B	109.1	C11—Pt1—C10	38.29 (16)
N1—C12—H12A	109.1	C11—Pt1—N1	93.39 (13)
N1—C12—H12B	109.1	C11—Pt1—Cl1	153.95 (12)
N1—C12—C13	112.6 (4)	N1—Pt1—Cl1	86.47 (8)
C12—C13—H13A	109.5		
			
C1—C2—C3—C4	−0.4 (6)	C9—C10—Pt1—Cl1	−49.7 (6)
C1—C2—C3—O1	−179.5 (4)	C10—C11—Pt1—C1	79.9 (2)
C1—C6—C9—C10	18.0 (5)	C10—C11—Pt1—N1	−97.9 (2)
C2—C1—C6—C5	2.1 (6)	C10—C11—Pt1—Cl1	173.18 (18)
C2—C1—C6—C9	−179.1 (4)	C11—C10—Pt1—C1	−96.0 (2)
C2—C1—Pt1—C10	167.0 (4)	C11—C10—Pt1—N1	85.9 (2)
C2—C1—Pt1—C11	128.9 (4)	C11—C10—Pt1—Cl1	−166.3 (4)
C2—C1—Pt1—Cl1	−25.0 (4)	C12—C13—C14—C15	55.2 (5)
C2—C3—C4—C5	1.4 (6)	C12—N1—Pt1—C10	−121.5 (3)
C2—C3—C4—O2	−178.8 (4)	C12—N1—Pt1—C11	−83.2 (3)
C2—C3—O1—C7	−7.9 (6)	C12—N1—Pt1—Cl1	70.7 (2)
C3—C4—C5—C6	−0.6 (6)	C13—C12—N1—C16	54.6 (4)
C3—C4—O2—C8	176.2 (4)	C13—C12—N1—Pt1	−172.1 (3)
C4—C3—O1—C7	173.0 (4)	C13—C14—C15—C16	−55.4 (5)
C4—C5—C6—C1	−1.2 (6)	C14—C15—C16—N1	55.6 (5)
C4—C5—C6—C9	−180.0 (4)	C15—C16—N1—C12	−54.4 (5)
C5—C4—O2—C8	−4.0 (6)	C15—C16—N1—Pt1	176.0 (3)
C5—C6—C9—C10	−163.2 (4)	C16—N1—Pt1—C10	8.4 (3)
C6—C1—C2—C3	−1.3 (6)	C16—N1—Pt1—C11	46.6 (3)
C6—C1—Pt1—C10	−11.7 (3)	C16—N1—Pt1—Cl1	−159.5 (3)
C6—C1—Pt1—C11	−49.8 (3)	N1—C12—C13—C14	−55.7 (5)
C6—C1—Pt1—Cl1	156.3 (3)	O1—C3—C4—C5	−179.5 (4)
C6—C9—C10—C11	51.5 (5)	O1—C3—C4—O2	0.4 (5)
C6—C9—C10—Pt1	−25.5 (4)	O2—C4—C5—C6	179.6 (4)
C9—C10—C11—Pt1	−100.7 (3)	Pt1—C1—C2—C3	−179.9 (3)
C9—C10—Pt1—C1	20.5 (3)	Pt1—C1—C6—C5	−179.1 (3)
C9—C10—Pt1—C11	116.6 (4)	Pt1—C1—C6—C9	−0.3 (5)
C9—C10—Pt1—N1	−157.6 (3)		

### Hydrogen-bond geometry (Å, º)

Cg1 is the centroid of the C1–C6 ring.

**Table:**

*D*—H···*A*	*D*—H	H···*A*	*D*···*A*	*D*—H···*A*
O3—H3···O1	0.84	2.10	2.869 (4)	152
O3—H3···O2	0.84	2.47	3.158 (4)	140
C10—H10···Cl1^i^	0.95	2.74	3.466 (4)	134
C7—H7*A*···O3^ii^	0.98	2.59	3.276 (6)	127
C15—H15*A*···*Cg*1^i^	0.99	2.68	3.572 (5)	149
C12—H12*A*···*Cg*1^iii^	0.99	2.61	3.529 (5)	154

Symmetry codes: (i) −*x*, −*y*+1, −*z*+1; (ii) −*x*+1, −*y*+1, −*z*; (iii) −*x*+1, −*y*+1, −*z*+1.

## References

[bb1] Bruker (2003). *SADABS*, *SAINT* and *SMART*. Bruker AXS Inc., Madison, Wisconsin, USA.

[bb2] Da, T. T., Chi, N. T. T., Van Meervelt, L., Mangwala Kimpende, P. & Dinh, N. H. (2015). *Polyhedron*, **85**, 104–109.

[bb3] Da, T. T., Kim, Y., Mai, T. T. C., Cuong, N. C. & Dinh, N. H. (2010). *J. Coord. Chem.* **63**, 473–483.

[bb4] Da, T. T., Minh, N. T. T., Chi, N. T. T. & Dinh, N. H. (2007). *Polyhedron*, **26**, 3271–3276.

[bb5] Darshan, S. & Doreswamy, R. (2004). *Phytother. Res.* **18**, 343–357. 10.1002/ptr.1475 15173991

[bb6] Dolomanov, O. V., Bourhis, L. J., Gildea, R. J., Howard, J. A. K. & Puschmann, H. (2009). *J. Appl. Cryst.* **42**, 339–341.

[bb7] Ford, R. A., Domeyer, B., Easterday, O., Maier, K. & Middleton, J. (2000). *Regul. Toxicol. Pharmacol.* **31**, 166–181. 10.1006/rtph.1999.1362 10854123

[bb8] Freeman, S. & Alder, J. F. (2002). *Eur. J. Med. Chem.* **37**, 527–539. 10.1016/s0223-5234(02)01382-x 12126772

[bb9] Groom, C. R. & Allen, F. H. (2014). *Angew. Chem. Int. Ed.* **53**, 662–671. 10.1002/anie.201306438 24382699

[bb10] Sheldrick, G. M. (2008). *Acta Cryst.* A**64**, 112–122. 10.1107/S0108767307043930 18156677

[bb11] Todd, E. S., James, E., Elaine, P., Suk, L. W. & Ritsuo, N. (2008). *Entomol. Exp. Appl.* **128**, 380–388.

